# Long-term safety and efficacy of frameless subthalamic deep brain stimulation in Parkinson’s disease

**DOI:** 10.1007/s10072-023-07059-2

**Published:** 2023-09-12

**Authors:** Danilo Genovese, Francesco Bove, Leonardo Rigon, Tommaso Tufo, Alessandro Izzo, Paolo Calabresi, Anna Rita Bentivoglio, Carla Piano

**Affiliations:** 1 Neurology Unit, IRCCS Fondazione Policlinico Universitario Agostino Gemelli IRCCS, Rome, Italy; 2 Department of Neurology, The Marlene and Paolo Fresco Institute for Parkinson’s and Movement Disorders, NYU Langone Health, New York, NY USA; 3 Department of Neuroscience, Università Cattolica del Sacro Cuore, Rome, Italy; 4 Neurosurgery Unit, IRCCS Fondazione Policlinico Universitario Agostino Gemelli IRCCS, Rome, Italy

**Keywords:** Deep brain stimulation, Frameless deep brain stimulation, Subthalamic nucleus, Parkinson’s disease

## Abstract

**Background:**

Bilateral deep brain stimulation (DBS) of the subthalamic nucleus (STN) is standard of care for Parkinson’s disease (PD) patients and a correct lead placement is crucial to obtain good clinical outcomes. Evidence demonstrating the targeting accuracy of the frameless technique for DBS, along with the advantages for patients and clinicians, is solid, while data reporting long-term clinical outcomes for PD patients are still lacking.

**Objectives:**

The study aims to assess the clinical safety and efficacy of frameless bilateral STN-DBS in PD patients at 5 years from surgery.

**Methods:**

Consecutive PD patients undergoing bilateral STN-DBS with a frameless system were included in this single-center retrospective study. Clinical features, including the Unified Parkinson’s Disease Rating Scale (UPDRS) in its total motor score and axial sub-scores, and pharmacological regimen were assessed at baseline, 1 year, 3 years, and 5 years after surgery. The adverse events related to the procedure, stimulation, or the presence of the hardware were systematically collected.

**Results:**

Forty-one PD patients undergone bilateral STN-DBS implantation were included in the study and fifteen patients already completed the 5-year observation. No complications occurred during surgery and the perioperative phase, and no unexpected serious adverse event occurred during the entire follow-up period. At 5 years from surgery, there was a sustained motor efficacy of STN stimulation: STN-DBS significantly improved the off-stim UPDRS III score at 5 years by 37.6% (*P* < 0.001), while the dopaminergic medications remained significantly reduced compared to baseline (− 21.6% versus baseline LEDD; *P* = 0.036).

**Conclusions:**

Our data support the use of the frameless system for STN-DBS in PD patients, as a safe and well-tolerated technique, with long-term clinical benefits and persistent motor efficacy at 5 years from the surgery.

## Introduction

DBS is an established treatment for movement disorders [1] and has become standard of care for patients with Parkinson’s disease [2], outperforming the best medical treatment for quality of life and management of motor symptoms in suitable patients [3]. DBS surgery has been historically performed using a stereotactic frame [4], entailing a resources- and time-consuming process and causing significant discomfort for patients. The frameless stereotaxy combines image-guidance technology and an advanced navigation system to curtail the surgery avoiding the frame placement, minimizing the preoperative planning [5], and reducing patients’ discomfort. This also corresponds to lower costs for frameless surgeries [6]. Despite this, the frame-based technique continues to be the most used in the vast majority of countries [7, 8].

Over the past decade, the frameless and frame-based systems have been extensively investigated by numerous groups and found to be equivalent in experimental and clinical accuracy [9–12]. Although several groups have investigated the topographical accuracy of the frameless stereotaxy [10], a few studies reported its clinical outcomes, mostly in the short term [13–15], with only one exploring the long-term in this population. In fact, our group reported clinical outcomes of patients undergone frameless STN-DBS for PD up to 3 years from implantation [16].

This retrospective study reports the 5-year clinical follow-up of PD patients who underwent bilateral STN-DBS with a manually adjustable frameless system.

## Material and methods

We carried out a retrospective analysis of PD patients who underwent bilateral STN-DBS in our center, IRCCS Fondazione Policlinico A. Gemelli in Rome, between 2012 and 2021. All the patients fulfilled the criteria for diagnosis of idiopathic PD according to the UK Parkinson’s Disease Brain Bank criteria [17] and the inclusion and exclusion criteria proposed by the core assessment program for surgical interventional therapies (CAPSIT) in PD panel [18]. Patients with previous neurosurgical interventions for PD or implantation of DBS electrodes in other deep brain nuclei were excluded from the study. Other exclusion criteria were dementia, epilepsy, and major active psychiatric disorder according to CAPSIT program [18].

### Surgical procedure

For each patient, contrast-enhanced volumetric T1-weighted 1.5 Tesla magnetic resonance imaging (MRI) of the whole head and T2-weighted images through the STN were obtained before the procedure. All patients underwent frameless surgery by using the NexFrame system (Medtronic®, Minneapolis, MN), with bilateral implantation of the DBS leads in the subthalamic nucleus, as detailed in a previous study [16]. The sensorimotor region of the STN was identified with single-track multipass microelectrode recording using 1 megaOhm platinum-iridium microelectrodes (FHC Corp, Bowdoinham, Maine). The number of tracks performed and track placement depended on the electrophysiological findings from the previous track. Unit recordings were performed with a Leadpoint 4 system (Medtronic, Minneapolis, MN). Repositioning of the electrode was considered in case of discrepancy between the expected length of the STN and the length obtained by MER or suboptimal MER signals. Intraoperative microstimulations were used to assess the therapeutic effect and side effect thresholds. Postoperative CT scans were obtained to rule out hemorrhage and to verify the lead location.

### Outcomes measures

The primary outcome of the study was to assess the clinical safety of frameless bilateral STN-DBS at 5 years from implantation. The secondary outcome was to assess the clinical efficacy of frameless bilateral STN-DBS at the 1-, 3-, and 5-year follow-up visits.

Patients’ demographic and clinical data collected at baseline and included in the analysis are shown in Table 1. The following variables were assessed at baseline (preoperatively), 1 year, 3 years, and 5 year after surgery:

- Score of the Unified Parkinson’s Disease Rating Scale (UPDRS) III and axial sub-score (items 27–31) [19], evaluated sequentially in the off-medication/on-stimulation, off-medication/off-stimulation, on-medication/off-stimulation, and on-medication/off-stimulation conditions.
- UPDRS part II in off-med state to assess the activities of daily living (ADL).
- UPDRS part IV to evaluate motor complications of the dopaminergic therapy; specifically, the items 32–34 were used as an overall assessment for dyskinesias and item 39 for daily off time.
- Hoehn and Yahr stage.
- Levodopa equivalent daily dose (LEDD; [20]).

**Table 1:** Patients’ demographic and clinical data at baseline, respectively of the entire population and of the patients completing the 5-year follow-up period. Data are shown as mean ± standard deviation

	Total sample	Patients completing the 5-year period
Number	41	15
Gender (male/female)	29–12	8–7
Age at intervention (years)	57.2 ± 7.4	57.1 ± 7.0
Disease duration (years)	11.8 ± 5.2	12.9 ± 6.3
Hoehn and Yahr stage	2.9 ± 0.6	2.8 ± 0.6
UPDRS III off-med	35.7 ± 11.4	32.2 ± 7.3
UPDRS III on-med	18.7 ± 6.6	16.7 ± 5.3
PD phenotype (AR-TD-MX)	26–11–4	9–4–2
LEDD (mg)	1263.8 ± 513.4	1080.6 ± 462.3

The first UPDRS motor score available was used to classify each patient into three motor subtypes: akinetic-rigid (AR), tremor-dominant (TD), and mixed subtype (MX); [21].

The efficacy of STN stimulation on motor symptoms was defined as the variation between the preoperative OFF medication condition and postoperative off-med on-stim condition. The efficacy of STN stimulation on axial symptoms was defined as the variation between the preoperative off-med axial sub-score and postoperative off-med on-stim condition axial sub-score of UPDRS. An adjunct evaluation of the current efficacy of DBS on motor symptoms was performed comparing postoperative UPDRS III score in off-med on-stim condition with off-med off-stim condition [22, 23].

Adverse events were systematically collected and classified as procedure-related, stimulation-related, and device-related.

### Data analysis

Continuous data comparing baseline and postoperative scores at 1-, 3-, and 5-year follow-ups were analyzed using the Wilcoxon signed-rank test. Statistical significance was set at *P* < 0.05.

All statistical computations were 2-sided and relied on Statistica 7.0 software (StatSoft, Tulsa, OK, USA).

## Results

**Fig. 1:**
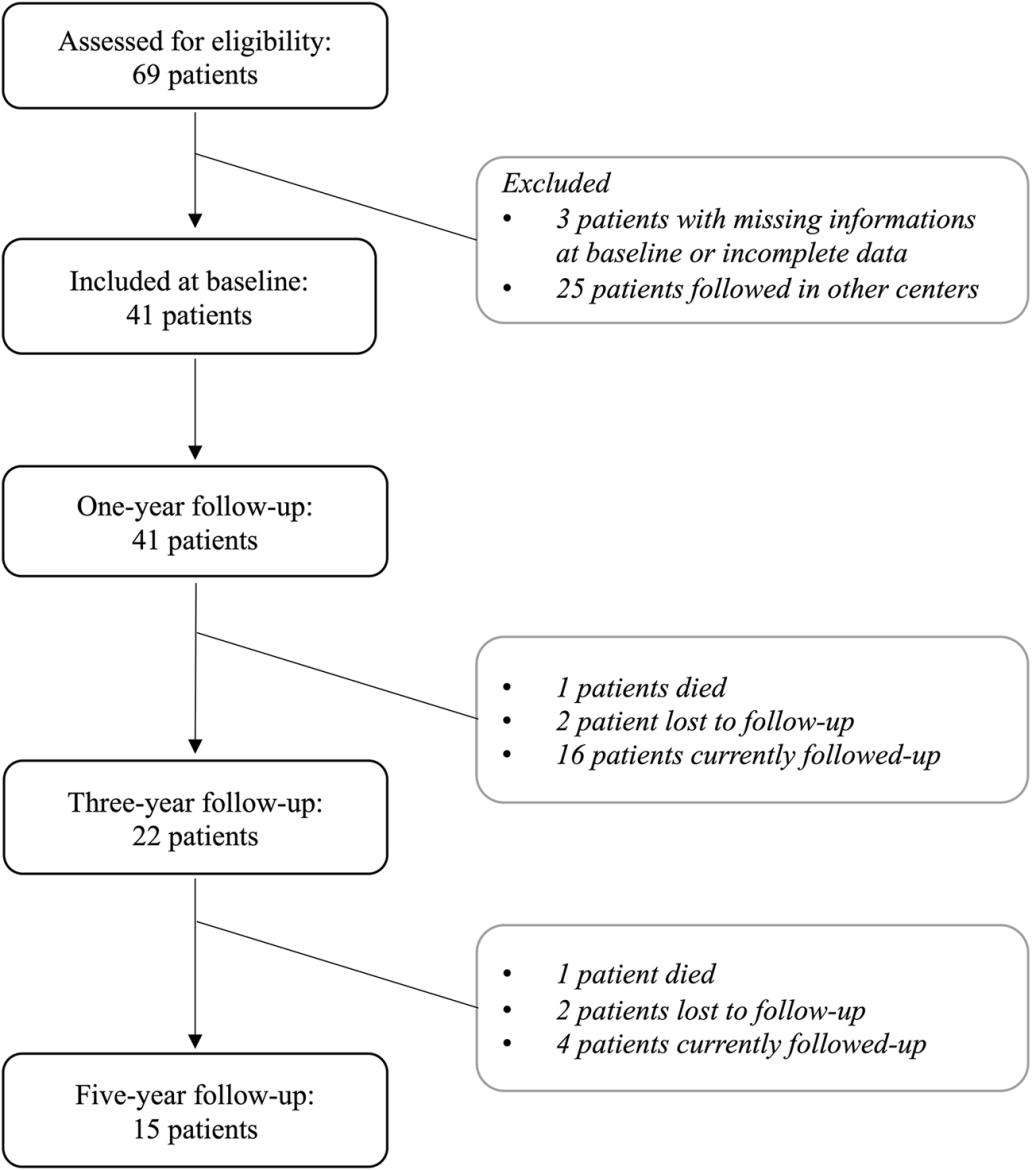
Study flow chart

Forty-one out of sixty-nine PD patients undergone bilateral STN DBS surgery between 2012 and 2021 followed at the Fondazione Policlinico Universitario A. Gemelli IRCCS in Rome (Italy) and were enrolled in the study (Fig. 1). Twenty-six of them (63.4%) presented with an akinetic-rigid phenotype, eleven patients (26.8%) had a tremor-dominant, and four patients (9.8%) had a mixed phenotype (Table 1). Their mean age at implantation was 57.2 ± 7.4 years with a mean disease duration of 11.8 ± 5.2 years and had a mean UPDRS III off-med of 35.7 ± 11.4 at baseline. MER tracks were performed per electrode for all the patients: in 69 out of 82 (84.1%) sides, the first track entered the sensorimotor region of STN. In the remaining recordings, two MER tracks were performed to optimize the STN targeting, except for two electrodes for which three MER tracks were needed. Fifteen out of the forty-one patients already completed the 5-year follow-up. Four patients were lost to follow-up and 20 subjects are currently being followed-up. Two patients died during the 5-year follow-up from causes unrelated to DBS (pneumonia and oral cavity neoplasm).

### Safety of frameless STN-DBS

No serious adverse events occurred during surgery or in the perioperative phase. During the 5-year follow-up, there was one device-related adverse event: the malfunction of one lead contact (increased impedances were found for one contact during a routine visit), which did not need interventions, and other contacts have been utilized since then. Among the stimulation-related adverse events, dysarthria was the most frequent motor side-effect, occurring in eleven cases. Two patients experienced involuntary eyelid closure (one for blepharospasm and one for apraxia of eyelid opening), treated successfully with botulinum toxin injections. Two patients had an isolated seizure after switching on the stimulation but never reoccurred with chronic stimulation. One patient developed asymptomatic peri-electrode edema in the postoperative phase, with regression of the radiological alterations within 2 weeks. The event neither required an intervention nor caused a prolongation of the hospitalization. One patient, a 61-year-old man with a multi-domain mild cognitive impairment before the intervention developed dementia at 2 years after implantation.

### Efficacy of frameless STN-DBS at 1- and 3-year follow-up

Forty-one patients completed the 1-year observation. The motor efficacy of STN stimulation in off-med condition was 30.1% in comparison with preoperative status (preoperative UPDRS III off-med 35.6 ± 11.4 versus UPDRS III off-med on-stim 24.9 ± 9.3, *P* < 0.00001). The UPDRS III axial sub-score was reduced by 38.7% (preoperative axial sub-score off-med 7.2 ± 3.8 versus axial sub-score off-med on-stim 4.4 ± 2.8, *P* < 0.00001). Likewise, STN-DBS reduced the dopaminergic regimen by 32.3% after 1 year (mean preoperative LEDD 1265.4 ± 519.8 mg versus 1-year LEDD 856.1 ± 476.3 mg, *P* < 0.0001).

Twenty-two patients completed the 3-year observation. The motor efficacy of STN stimulation in off-med condition was 25.3% in comparison with preoperative status (preoperative UPDRS III off-med 33.7 ± 8.0 versus UPDRS III off-med on-stim 25.1 ± 10.9, *P* = 0.00208). The UPDRS III axial sub-score was reduced by 22.8% (preoperative axial sub-score off-med 6.9 ± 2.5 versus axial sub-score off-med on-stim 5.3 ± 3.5, *P* = 0.0251). STN-DBS decreased the dopaminergic medications by 25.3% at 3 years (preoperative LEDD 1123.1 ± 449.1 mg versus 3-year LEDD 839.3 ± 394.0 mg, *P* = 0.01174).

### Efficacy of frameless STN-DBS at 5-year follow-up

**Fig. 2:**
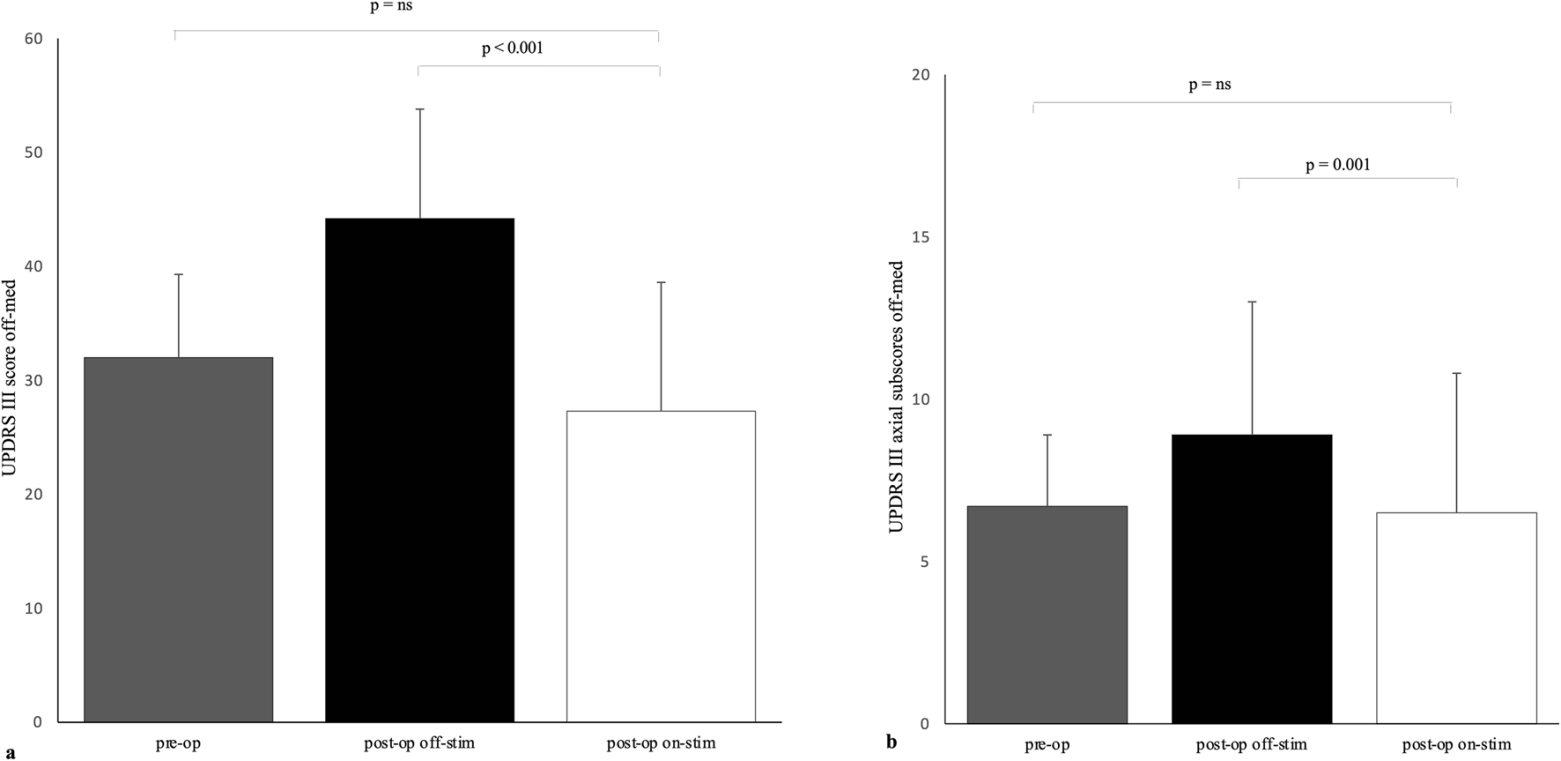
Comparison UPDRS III at 5-year visit vs baseline. **a** Preoperative off-medication versus postoperative off-medication off-stimulation versus postoperative off medication on-stimulation UPDRS III scores. **b** Axial subscore UPDRS 5-year visit. Preoperative off-medication versus postoperative off-medication off-stimulation versus postoperative off-medication on-stimulation UPDRS III axial subscores

**Fig. 3:**
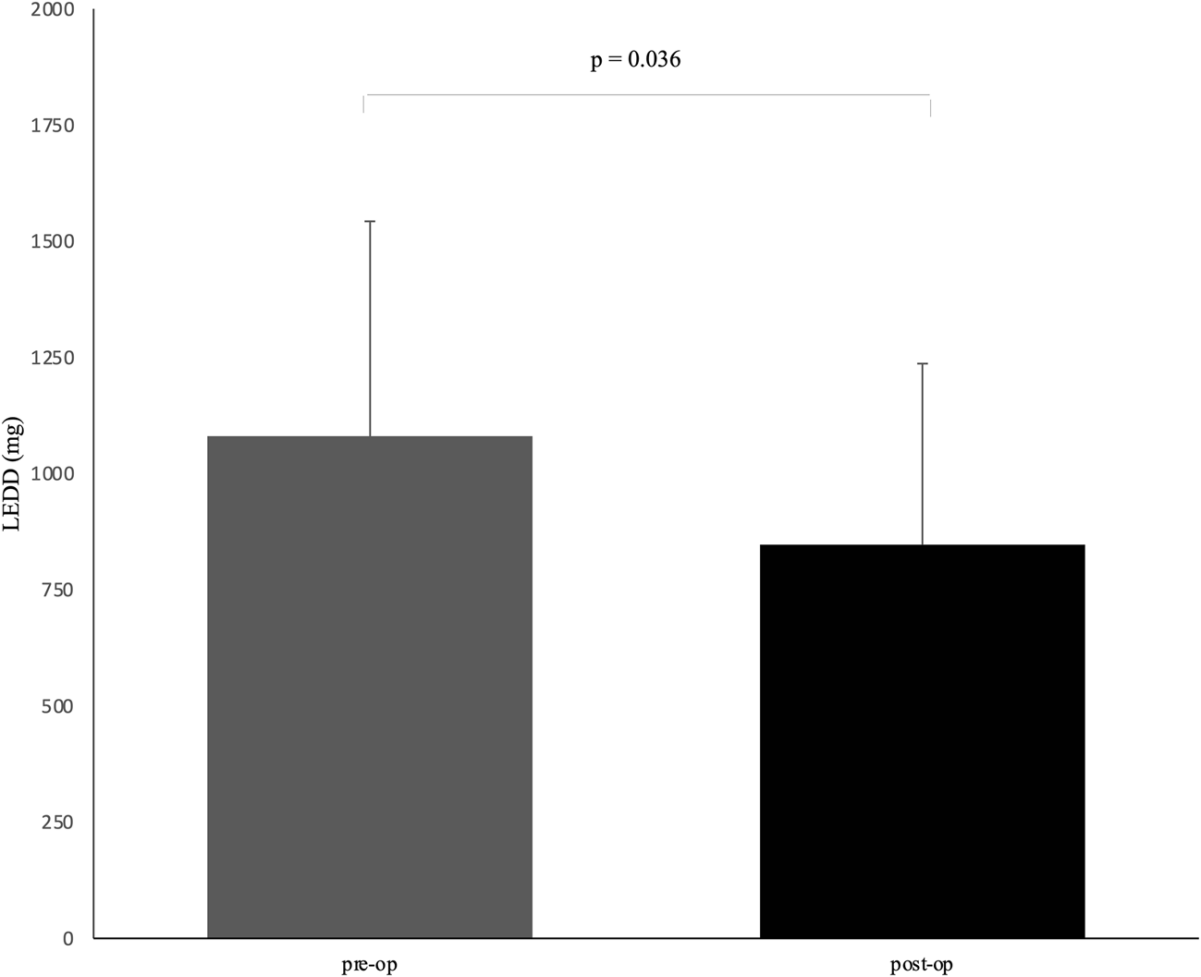
LEDD 5-year visit vs baseline. Preoperative versus postoperative levodopa equivalent doses (LEDD)

Fifteen patients completed the 5-year observation (Table 1). The motor efficacy of STN stimulation in off-med condition compared with preoperative status was 15.1%, without reaching the statistical significance (preoperative UPDRS III off-med 32.2 ± 0.6 versus postoperative UPDRS III off-med on-stim 27.3 ± 11.3, *P* = 0.25), while it was 37.6% when comparing the on-stim with the off-stim condition at 5-year follow-up (UPDRS III off-med off-stim 44.2 ± 9.6 versus UPDRS III off-med on-stim 27.6 ± 11.7; *P* = 0.00096). One patient did not tolerate the off-stimulation condition (Fig. 2a). Axial sub-score did not differ from the preoperative condition (preoperative axial UPDRS sub-score off-med 6.7 ± 2.2 versus axial UPDRS sub-score off-med on-stim 6.5 ± 4.3, *P* = 0.7795), but STN-DBS significantly improved it by 31.9% in comparison with the off-med off-stim condition (postoperative axial sub-score off-med off-stim 8.9 ± 4.1 versus axial UPDRS sub-score off-med on-stim 6.1 ± 4.4, *P* = 0.00148; Fig. 2b). After 5 years from DBS, dopaminergic drugs were significantly reduced by 21.6% compared to the baseline (preoperative LEDD 1080.6 ± 462.3 mg versus postoperative LEDD 847.2 ± 389.5 mg, *P* = 0.0357; Fig. 3).

### Activities of daily living

ADL in the off-medication state (UPDRS-II) improved with DBS at the 1- and 3-year follow-up, respectively, by 40.8% (preoperative UPDRS II score 16.7 ± 2.6 versus 1-year UPDRS II score 9.9 ± 3.3; *P* < 0.00001) and 24.4% (preoperative UPDRS II score 16 ± 2.2 versus 3-year UPDRS II score 12.1 ± 3.4; *P* < 0.00138). At 5 years, there was a non-significant reduction by 12.9% of the baseline score (preoperative UPDRS II score 16.1 ± 2.3 versus 5-year UPDRS II score 14.0 ± 3.6; *P* = 0.099).

### Motor complications: dyskinesias and daily off time

There was a sustained improvement in UPDRS IV total score throughout the entire follow-up period after surgery. It diminished by 68.9% at 1 year (preoperative UPDRS IV score 7.4 ± 3.1 versus 1-year UPDRS IV score 2.3 ± 1.9; *P* < 0.00001), by 56.3% at 3 years (preoperative UPDRS IV score 6.9 ± 2.1 versus 3-year UPDRS IV score 3.0 ± 1.8; *P* = 0.001), and by 33.3% at the 5-year follow-up (preoperative UPDRS IV score 7.5 ± 1.8 versus 5-year UPDRS IV score 5.0 ± 2.1; *P* = 0.01). Specifically, dyskinesias improved by 79.4% at 1 year (preoperative UPDRS IV items 32–34 score 3.4 ± 2.3 versus 1-year UPDRS IV items 32–34 score 0.7 ± 1.0; *P* < 0.00001), 65.4% at 3 years (preoperative UPDRS IV items 32–34 score 3.1 ± 1.7 versus 3-year UPDRS IV items 32–34 score 0.9 ± 1.1; *P* = 0.006), and 62.1% at 5 years from surgery (preoperative UPDRS IV items 32–34 score 3.3 ± 1.5 versus 5-year UPDRS IV items 32–34 score 1.2 ± 1.5; *P* = 0.025), compared to baseline. Daily off time diminished by 72.8% at 1 year (preoperative UPDRS IV item 39 score 1.7 ± 0.6 versus 1-year UPDRS IV item 39 score 0.5 ± 0.6; *P* < 0.00001), 62.2% at 3 years (preoperative UPDRS IV item 39 score 1.6 ± 0.6 versus 3-year UPDRS IV item 39 score 0.6 ± 0.5; *P* = 0.0015), and 49.8% at 5 years (preoperative UPDRS IV item 39 score 1.7 ± 0.6 versus 5-year UPDRS IV item 39 score 0.8 ± 0.5; *P* = 0.005).

## Discussions

This study represents the longest clinical follow-up, presenting data up to 5 years from surgery, for PD patients undergone bilateral subthalamic DBS with a frameless approach. In this study, we reported excellent results in terms of safety, as we did not have major surgery-related adverse events. There were no infections or suicide attempts during the 5-year follow-up. Only minor stimulation-related side effects, none of these definable as unexpected DBS-related adverse event, and two non-severe device-related adverse events (malfunction of one lead contact and peri-electrode edema) occurred during the follow-up period. The median proportion of surgery-related adverse events for STN-DBS ranges from 1 to 4%, as reported in STN-DBS literature to date. The median proportion of surgery-related was 3.45% for intracranial hemorrhage (ICH), of which 1.8% with permanent neurological deficits, while for surgery-related infections was 5.1%. The median proportion of hardware-related events reported was 3.96% for late not surgery-related infections, 4.1% for IPG dislocations, and 2.8% for lead rupture [2]. We did not report any of these events in our group of patients; however, the considerably larger amount of data available for frame-based DBS calls for precaution when comparing these studies with ours [2, 24, 25]. The prevalence of dysarthria observed in our population after DBS is in keeping with previously published data, where it varies between 1% after 6 months and 70% at 3 years, with an average of about 10% at 1 year from surgery [2, 26, 27].

In our population, STN-DBS significantly reduced the baseline motor UPDRS at the 1- and 3-year follow-up (by 32.1% and 25.3%, respectively). At 5 years from the surgery, the motor benefit of STN-DBS in comparison with the baseline condition did not reach the statistical significance. However, pondering the underlying disease progression, thus comparing the postoperative off-med on-stim with the postoperative off-med off-stim UPDRS III scores at 5 years from the surgery, we observed a sustained motor efficacy of DBS. Long-term follow-up studies have proven a significant improvement of the baseline UPDRS III up to 5 years after a frame-based DBS implantation, and few studies even beyond that time [23, 28–30]. The small size of the sample might have hampered the statistical significance of our 5-year results and substantial differences regarding the patients’ characteristics of the previous long-term studies merit consideration too: the patients included in those studies had a high preoperative off-med UPDRS III score [31] ranging from 50.2 to 59.5 (substantially stable during follow-up) [23, 28, 29], while our patients completing the 5-year follow-up had a significantly lower preoperative off-med UPDRS III, of 32.2 ± 7.3, and higher preoperative motor UPDRS off-medication condition is known to correlate with a later reduction of DBS efficacy [26, 32, 33]. Furthermore, in the long-term frame-based DBS studies, the preoperative levodopa response ranged between 55.0 and 71.1% at baseline [23, 28–30], while our patients demonstrated a mean improvement of UPDRS III scores on-medication of 48.1% at baseline, and the preoperative levodopa responsiveness is known to be strong predictor of the STN-DBS motor responsiveness, at least in the short term [2, 33–35]. Therefore, extending the indication for DBS to PD patients with milder motor impairment in line with recent evidence [1] and most current tendency [36], likely contributed to the earlier apparent reduction of motor benefit from DBS. Hence, the different disease severity at baseline might explain the different trend in our population, being the curve of disease progression steeper in milder cases [37], as well as in rigid-akinetic phenotype [33] that is clearly predominant in our population. On these grounds, our study reports data on efficacy of the frameless STN-DBS in line with the current frame-based DBS literature during the 5-year observation.

As previously reported [23, 28, 29, 38], we observed a smaller benefit of STN stimulation on axial symptoms of PD, which tended to significantly decline at 5 years. After 5 years from surgery, the axial symptoms did not differ from the baseline, even though we detected a persistent significant beneficial effect of STN-DBS on axial symptoms when comparing the on-stim with the off-stim condition.

Concomitantly, we observed a persistent reduction of dopaminergic therapy by 34.2%, 25.3%, and 21.6%, respectively, at 1, 3, and 5 years after implantation, compared with baseline LEDD. In large randomized-controlled studies with frame-based stereotaxy, during 6 to 12 months long follow-up, the levodopa equivalent dosage was reduced by 23–50% [1, 39–41] with longer-term follow-up studies showing a reduction of baseline LEDD up to 5 years from the surgery, similarly to our study [23, 28].

Bilateral STN-DBS also resulted in a significant reduction of UPDRS-II with improvement of the ADL in our population, although the benefit of STN-DBS gradually declined over the years and did not reach the statistical significance at 5 years. This is plausibly due to the disease progression with worsening of dopa-resistant motor and non-motor symptoms, as described in previous studies [42, 43], and sample size. Our data are in line with the most recent DBS literature, showing lower UPDRS-II at baseline than in previous studies, with an apparent milder benefit of DBS on the UPDRS-II score. In fact, compared to the period covered by Kleiner-Fisman et al. (1993–2005) [26], the STN studies since 2005 had shown lower scores at baseline and slightly lower response of UPDRS-II to DBS [2], most likely due to the shorter disease history and lower severity at baseline of the subjects enrolled.

In addition, we observed a sustained benefit of STN-DBS on motor complications throughout the entire follow-up period after surgery: dyskinesias severity and duration remained markedly reduced, as well as the daily-off time at 5 years from surgery. Data regarding motor fluctuations and dyskinesias are still very heterogeneous in the literature and, as a result, difficult to compare between studies, due to the use of different evaluation tools (UPDRS items in different combinations, patient diaries or other rating scales) and missing reporting [2]. However, the efficacy of DBS on motor complications was maintained in the long-term follow-up, as expected [44]. This is particularly relevant to PD patients, considering the great impact of motor complications on ADL and quality of life [45].

Hence, this study shows the excellent safety profile and good and sustained clinical efficacy over time of frameless DBS. Along with the several pros for patients, decreased MER time, fewer trajectories with shorter operative time, and costs observed in the frameless approach are additional advantages for clinicians and health system [5, 6]. Despite this, the frame-based technique is largely the most used in many countries [7, 8].

While the strength of this study is the length of follow-up for this specific population, some limitations need consideration. The frameless system is the only technique available for DBS implantation at our center since 2012 and we could not compare it with a frame-based DBS control group. Furthermore, being the study monocentric, the relatively small sample size of our population limited the statistical significance of some long-term results. The third limitation is the lack of specific scales on quality of life due to the retrospective nature of our study. Future prospective controlled studies are advisable to corroborate our findings.

## Conclusions

Bilateral STN-DBS, using frameless stereotaxy and single-track multipass MER, shows an excellent safety profile, and results in long-term good motor outcomes for PD patients, with persistent benefits at 5 years from surgery. Our data, together with the considerable advantages for patients, clinicians, and health system, support the use of the frameless system for DBS of PD patients.

## Data Availability

Anonymized data of this study will be available from the corresponding author upon reasonable request from any qualified researcher, following the EU General Data Protection Regulation.
